# Study on Tripping Risks in Fast Walking through Cadence-Controlled Gait Analysis

**DOI:** 10.1155/2018/2723178

**Published:** 2018-05-24

**Authors:** Wen-Fong Wang, Wei-Chih Lien, Che-Yu Liu, Ching-Yu Yang

**Affiliations:** ^1^Department of Computer Science and Information Engineering, National Yunlin University of Science and Technology, Douliu, Yunlin 640, Taiwan; ^2^Department of Physical Medicine and Rehabilitation, National Cheng Kung University Hospital, College of Medicine, National Cheng Kung University, Tainan 600, Taiwan; ^3^Department of Computer Science and Information Engineering, National Penghu University of Science and Technology, Magong, Penghu 880, Taiwan

## Abstract

Fast walking is a common exercise for most people to promote health. However, a higher cadence due to fast walking on ordinary or uneven ground raises the risk of tripping. To investigate the tripping issue, research to observe the gait in fast walking is needed. To explore the relationship between fast gait and the risk of tripping, a gait recording system with a specific synchronization mechanism was developed in this work. The system can acquire gait signals from wearable sensors and action cameras at different cadences. Meanwhile, algorithms for gait cycle segmentation and characteristic extraction were proposed for analyzing a fast gait. In the gait analysis, the correlations of low, moderate, and high cadence in cueing and no cueing gaits were computed, and two results were obtained. First, the higher the cadence is, the larger the motion strength in the terminal foot swing will be and the smaller the motion strength at the starting foot swing. Second, the decreased distance of foot clearance becomes more conspicuous as the cadence increased, especially if one is walking more than 120 beats. The results indicate that fast walking with bigger strides and lower cadence is the best way to maintain safety in moving over ordinary ground.

## 1. Introduction

Walking is an important daily activity, and many people do that to maintain healthy condition. Movements in walking can be affected by the rhythmic sounds, which make the movements smoother and more stable [1, 2]. In walking, many people use the music tempo to regulate their cadence and breathing rate to keep their exercises more lasting. Additionally, they also conduct fast walking to increase their exercise intensity for calorie consumption. In terms of medical treatment, many patients that have Parkinson's disease [3], children with cerebral palsy [4], and many patients that have strokes [5] and gait rehabilitation [6] use metronomes to help their exercises.

In the literature, rhythm on gait has two research topics, which are in health promotion and medical applications. For the health promotion, some studies reveal that the music tempo affects gait movements and benefits human health conditions. In [7], Mendonca et al. studied the music played at the treadmill to regulate the walking pace and analyze the gait parameters. Their experiment included walking with and without music cueing synchronization, and the results showed that when there was music cueing synchronization, people would change their walking step with the music tempo. When there was no music cueing synchronization, people would not change their walking step with the music tempo obviously. In [8], Eikema et al. found that old people's gait parameters, such as the stride and step, reduced dramatically, and their walking speed could be changed using the voice cue to regulate their pace on treadmills. After the training on walking in cue, their gait and strides were improved obviously to be closer to young people.

In medical applications, acoustic tempos are frequently used in gait rehabilitation, with positive instantaneous and prolonged transfer effects on various gait characteristics. In [9], rhythmic auditory cues including music and metronome beats were used to improve disordered gait in clinical conditions. Their study indicated that music and metronome cues may not be used interchangeably, and the cue type and frequency (cadence) should be considered when evaluating effects of rhythmic auditory cues on gait. In [10], the gait pattern in various rhythmic auditory stimulation tempos was investigated in stroke patients. The results showed that as the tempo increased, the spatiotemporal gait parameters of the stroke patients changed significantly. After gait training with rhythmic auditory stimulation, gait parameters of the affected and unaffected sides improved significantly compared to baseline.

In the previous studies, treadmills were often used for step and pace control, and thus, the conducted experiments were often restricted in space [5, 7, 8, 10]. Therefore, the research results were biased due to few data from daily walk. In recent years, the advancement of the wearable technology has made sensors miniaturized and widely applied in many research and applications [11]. As the wearable sensors overcome space restrictions and improve the degree of freedom in use, these devices can be used to replace fixed and expensive measuring apparatuses [12]. In this study, we developed a gait recording system, which equipped with a set of wearable triaxial acceleration sensors and action cameras. The sensors are in miniature size and can be easily worn by subjects to acquire gait signals from daily walk. The action cameras can be used to record gait movements. With this system, gait signals for daily walking can be acquired without space restrictions.

In [13], Rubenstein described that the risks of falls and trips are due to some identifiable factors such as weakness, unsteady gait, confusion, and certain medications and, simultaneously, indicated that attention to the factors can greatly reduce the rates of falling. He also described that one of the most effective fall reduction programs involve environmental inspection and hazard reduction. In [14], Lord et al. indicated that the most challenging area to incorporate into fall aetiology is that of environmental risk factors since the research base is smaller than that for other types of risk factors. At the same time, research in this area has typically been less rigorous or has been troubled by methodological limitations. Since a gait recording system with the wearable technology has been developed in this work to improve the methodological limitations, we are interested in exploring the tripping risks in fast walking through an uneven ground, which may exist with a few environmental risk factors, by a cadence-controlled strategy. With the application of action cameras and a specific synchronization mechanism between the signals from the wearable sensors and the images from the cameras, we realize the gait analysis of fast walking without space restrictions.

This investigation is organized as follows: Section 2 presents research materials, including gait description, gait signal acquisition, cadence control, and a signal synchronization mechanism.  Section 3 describes research methods, including the experimental protocol, signal preprocessing, signal segmentation and characteristic extraction, and correlation analysis.  Section 4 shows the results, which include correlation analysis in cadence versus gait events and periods with and without cueing. In addition, analyses of gait phase variation and gait periods versus walking types are also done for exploring the tripping risks. Concluding remarks and future work are given in Section 5.

## 2. Research Materials

### 2.1. Gait Description

**Figure 1:**
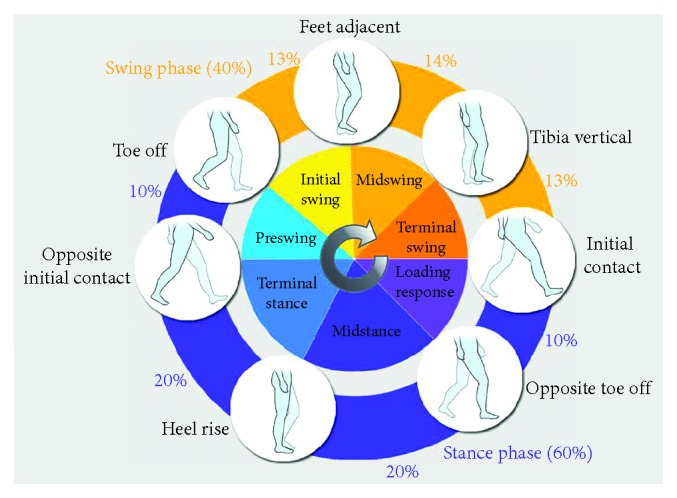
Events, periods, and cycles of human gait [15].

Human walking consists of a number of cyclically sequential gait events, and one complete gait cycle (Figure 1) can be measured from one toe off event to the next one [15]. As shown in Figure 1, a full gait cycle includes a stance phase and a swing phase, where 60% of the cycle is spent in stance and 40% in swing [16].

The gait cycle has seven gait events, that is, initial contact (IC), opposite toe off (OTO), heel rise (HR), opposite initial contact (OIC), toe off (TO), feet adjacent (FA), and tibia vertical (TV) [17]. It can also be subdivided into seven periods, that is, loading response, midstance, terminal stance, preswing, initial swing, midswing, and terminal swing. While finely subdividing gait events and periods for every cycle, different gait parameters such as the cycle duration, stance time, swing time, stride length, step length, cadence, and walking speed can be analyzed to learn about personal gait features.

### 2.2. System of Signal Acquisition

**Figure 2:**
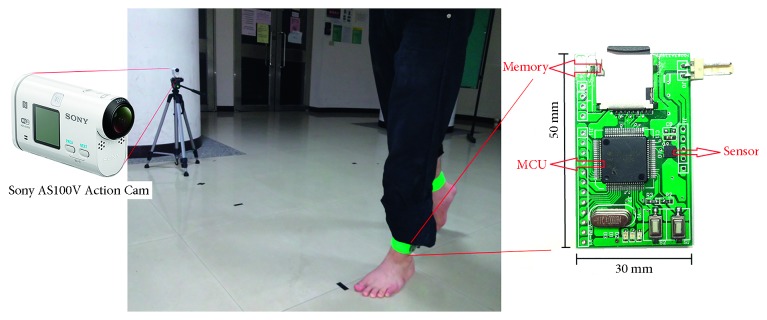
Wearable sensor module with a triaxial accelerometer and a high-speed camera for gait signal acquisition.

In this study, a wearable sensor module with a triaxial accelerometer shown in Figure 2 was developed to acquire motion signals related to human gait. The size of this sensor is about 50 × 30 mm^2^, and it includes a microprocessor (TI MSP430™ series [18]), a triaxial accelerometer (ST LIS3DH [19]), a 8 GB micro-SD flash memory card, and a 3.3 V lithium battery. The acceleration measuring range of the sensor is dynamically user selectable full scales of ±2  g/±4  g/±8  g/±16  g, and the sampling data rate is programmable from 1 Hz to 5 kHz. Here, the acceleration measuring range is set to ±16  g and the sampling data rate to 120 Hz. For the acquired signal data, they would be stored in the micro-SD memory card temporarily, and later, the data are uploaded to server computers for signal processing. In the procedure of motion signal acquisition, the sensor would be attached steadily on one foot of a subject to a position 2∼3 cm above his/her ankle.

To record the video of gait movements, a camera (Sony AS100V Action Cam [20]) shown in Figure 2 was applied. The highest image capture rate of the camera is 240 FPS (frames per second), and each captured image has the resolution of 1280 × 720 pixels. Thus, the camera satisfies the requirement of our experiment for recording and watching the actions of gait events. For the sake of recording synchronization between the sensors and cameras, the image capture rate was set to 120 FPS to meet the requirement of synchronizing gait signals and foot locomotion images.

### 2.3. Control of Cadence

In this study, the music tempo was employed to adjust the cadence of subjects for observing the gait variability in fast walking. Normally, the music tempo is calculated by beats per minute (BPM), and the number of beats for walking means the number of steps to go in a minute. Generally, the cadence of a normal person in walking is about 80∼120 BPM, and for fast walking, it is about 120∼140 BPM. For the purpose of comparing the gait patterns in slow, normal, and fast walking, we focused on the tempo of 80, 100, and 120 BPM for the controlled cadence.

Usually, music has many different styles such as pop music like hip-hop, rock and roll, and jazz, which have a strong tempo to ease the regulation of the walking rhythm. Here, the jazz's tempo was considered to regulate the walking rhythm [21]. This idea has three considerations. Firstly, it usually possesses the drum sounds of low frequency for beats so that the subjects can identify them clearly while walking. Secondly, the jazz's tempo is almost fixed and good for gait control. Lastly, it can make subjects feel relaxed and walk with ease. Although a metronome seems a little better than the jazz's music to provide a clear tempo, we still preferred the jazz's tempo due to the above considerations.

### 2.4. Synchronization between Motion Signals and Captured Images from Video

In the aforementioned signal acquisition system, the sampling rate of the wearable sensors was set to 120 Hz, and the video frame rate of the high-speed cameras was also set to 120 FPS. Therefore, a synchronization mechanism is needed to relate the image of one gait event recorded by the camera to one motion signal acquired by the wearable sensor sequentially for identifying the gait characteristics. To complete the synchronization, a leading clearance matching synchronization mechanism was proposed, and we asked all participants to stand still for 10 seconds after the setup of the sensors and cameras and before each walking test. In this way, a long and clear section of the temporal gap can be created for identifying the synchronized origin of the gait images and motion signals.

**Figure 3:**
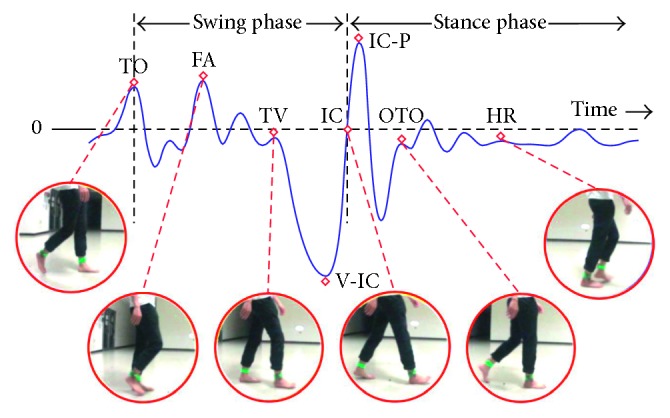
Matching gait characteristics for the gait events of the right foot through image marking in one gait cycle.

Under the supervision of one medical expert in our team, we discovered the gait events continuously in the recorded video through a frame-by-frame video player in slow motion. At the same time, we marked the temporal position of each found image and used that position to extract the corresponding accelerometrical signal of each gait event in the acquired signal set. In this study, we call the corresponding accelerometrical signal of a gait event as a gait characteristic. As shown in Figure 3, a sample curve of gait cycles acquired from the right foot of one participant is depicted with a significant valley (labeled by “V-IC”), a significant peak (“IC-P”), and six gait events. For the gait characteristics of the left foot, they can be acquired in the same way as above. By locating several sets of the gait cycles as a reference and using specific computer algorithms, it becomes easy and quick to find out the gait events in the recorded video and the corresponding gait characteristics.

Through the synchronization mechanism, the appeared gait characteristics can be verified exactly by the actions of the gait events as shown in Figures 1 and 3. From the temporal positions of the gait events and characteristics, relevant gait parameters can be calculated accordingly.

## 3. Research Methods

**Figure 4:**
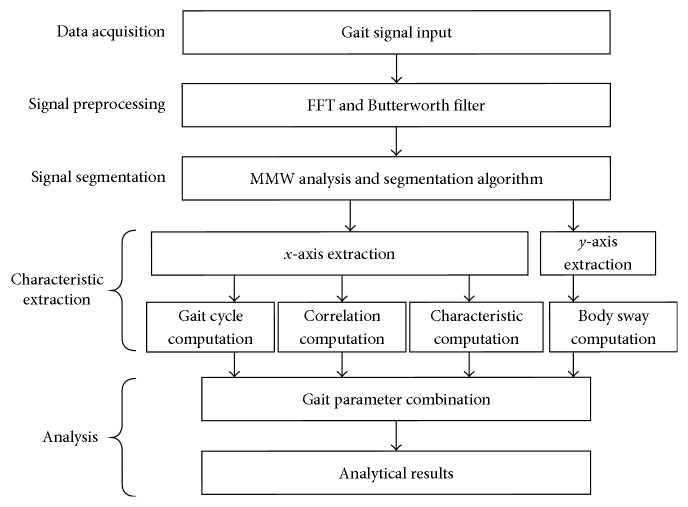
The flow diagram of gait signal processing.

To obtain useful human gait information, there are five stages, that is, data acquisition, signal preprocessing, signal segmentation, characteristic extraction, and analysis, for the signal processing of gait characteristics (Figure 4). For the gait signal acquisition, two wearable triaxial acceleration sensors were used, where each foot was put on one sensor. Since there were many other unrelated signals involved in the raw gait signals from the sensors, the fast Fourier transform (FFT) [22] was used to analyze the noise and signal spectra and a Butterworth filter [23] to remove the noise. Subsequently, the filtered signals on *x*- and *y-*axes were segmented according to the gait cycles with the assistance of using the modulus maxima wavelet (MMW) analysis [24] to identify the temporal separation positions. A number of gait characteristics were then extracted to compute the essential gait parameters. Finally, the gait parameters were fused together for the gait analysis of tripping risks in free and fast walking under various cadences regulated by different jazz's tempos.

### 3.1. Experimental Protocol

In this study, twenty-five healthy participants were recruited for the experiment of walking under different cadences, and each of them had completed an auditory test to ensure that their hearing and tempo recognition were normal. In terms of performing all gait movements, all participants had been verified to have no medical restrictions on their activities, tempo recognition, and walking. Before the walking tests, all participants only knew that they would take part in the experiment of normal walking, and they did not know that the experiment involved musical rhythm. The data obtained from the cases of without and with playing music tempos would be compared and then be analyzed for the effects of walking on playing tempos for all gait events and periods.

**Figure 5:**
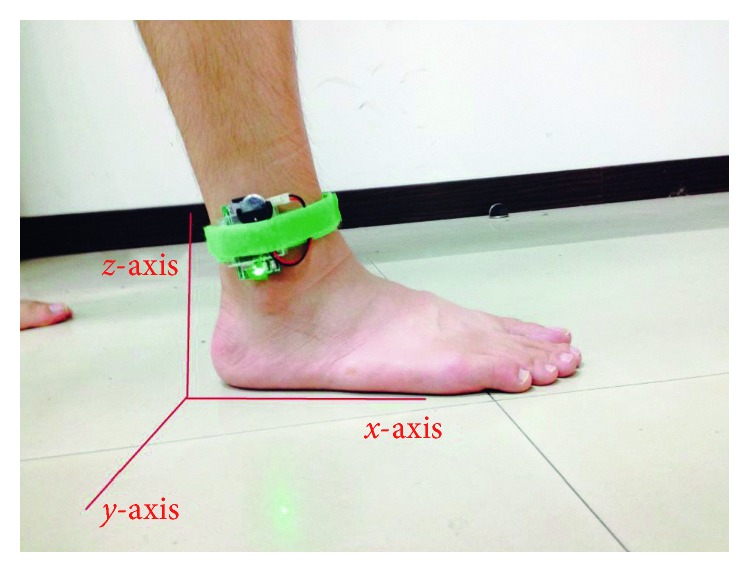
The 3D coordinate system for signals from the wearable triaxial acceleration sensors.

In the walking tests, the sensors were fixed on the feet of the participants at the position 2∼3 cm above their ankle of each foot. The sensors measure the gait signals in a 3D coordinate system, where the longitudinal, lateral, and vertical axes are represented as *x*-, *y*-, and *z*-axes as shown in Figure 5. It is worthy to note that the signal component of the longitudinal axis (or *x*-axis) has the most obvious features of gait while walking and shows mainly the movement characteristics in the acceleration aspect. Therefore, the *x*-axial component of the gait signals in the accelerometer was mainly investigated and analyzed to extract the gait parameters in our study. For the signal's lateral axis (or *y*-axis), the corresponding signal components reveal the left and right accelerations of gait movements, and they help to understand the walking balance of participants. Thus, the *y*-axial signal components of gait movements would be incorporated into our study of gait analysis. In the observations of the signal's vertical axis (or *z*-axis), it was found that the signal's variations were greatly with personal habit in walking, and it might be suitable to investigate personal walking habit. Therefore, the signal components of *x*- and *y*-axes were considered as the basis to study and analyze the gait characteristics of tripping risks in this study.

**Figure 6:**
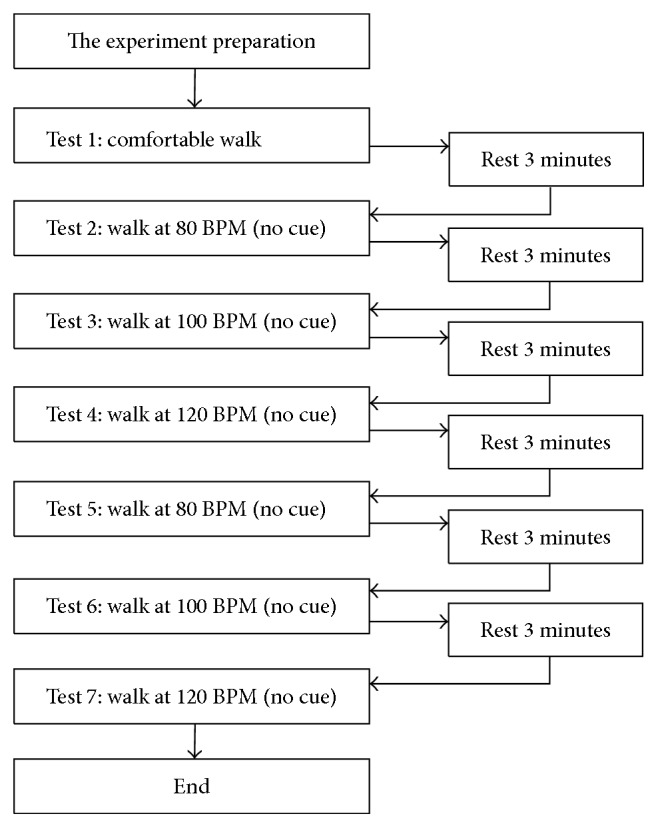
The flow diagram of the walking tests.

After installing the sensors, the participants were asked to walk barefoot to and fro the experimental pathway for 3 minutes so that they could get accustomed to walking naturally wearing the sensors. In the tests, all participants walked barefoot on a 30-meter straight pathway of a ceramic tile floor in 7 different walking tests, which followed the procedure as shown in Figure 6. For the purpose of synchronizing the motion signals and captured images from video, one camera installed at the starting point of the pathway was employed. In addition, there were six cameras set on both sides of the pathway at the positions of 10, 15, and 20 meters from the starting point. These cameras were used to record the gait movements in the tests.

For the first test, the participants were asked to walk in their most natural and comfortable way, which would be treated as the normal gait (Test 1). After that, they were asked to take a 3-minute rest, and meanwhile, jazz music at 80 BPM was played to prepare the participants for the next test. The volume of the music that the participants listened to was around 65∼70 db, since the volume of conversation between normal people is usually about 60 db. After the rest, the participants were asked to walk while the jazz at 80 BPM was playing (Test 2), and then, they were asked to take the second 3-minute rest with the jazz at 100 BPM being played. After that, they were asked to walk while the jazz at 100 BPM was playing (Test 3), and then, they took the third 3-minute rest with the jazz at 120 BPM being played. After that, they walked again while the jazz at 120 BPM was playing (Test 4), and then, they took the fourth 3-minute rest with the jazz at 80 BPM being played. Up to this point, the participants completed the tests with no cue.

For the subsequent tests, the participants were asked to listen to the played jazz's tempo and synchronize their walking cadence with the tempo. The fourth 3-minute rest could enable them to adapt their walking cadence to follow the music tempo. The next test made the participants walk to the beats of the jazz at 80 BPM (Test 5). After that, they took the fifth 3-minute rest with the jazz at 100 BPM being played. After the rest, they were asked to walk to the beats of the jazz at 100 BPM (Test 6). Then, after the sixth 3-minute rest with the jazz at 120 BPM being played, they were asked to walk to the beats of the jazz at 120 BPM (Test 7).

### 3.2. Signal Preprocessing

**Figure 7:**
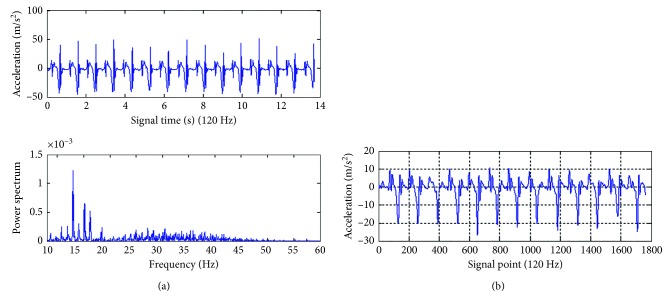
Gait noise removal: (a) the raw gait signals and their FFT spectrum and (b) the filtered gait signals.

Since the sensors have very high sensitivity, slight environmental vibrations may cause significant and abrupt changes in the acquired signals. As the gait signals were captured and recorded by the sensors, the original signals obtained were often mixed up with many other irrelevant signals, which are collectively called noises. As a consequence, the FFT was employed to find out the major spectrum of the gait movements of common people as shown in Figure 7(a). It can be seen that the frequency distribution is roughly from 0 Hz to 50 Hz. Since the gait movements of common people are usually at a lower frequency band, the Butterworth filter [25], which is a low-pass filter and has high computational efficiency, was used to remove the frequency higher than 20 Hz for the gait analysis in this study. After removing the noise signals, the filtered gait signals can be seen as shown in Figure 7(b).

### 3.3. Gait Cycle Segmentation and Characteristic Extraction

**Figure 8:**
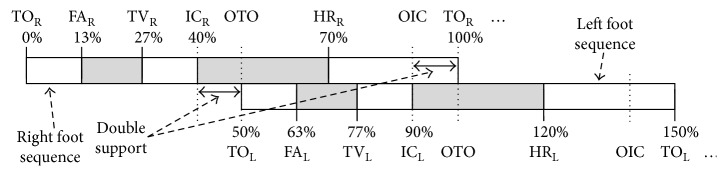
Gait signal synthesis of the left and right feet: the cyclic sequence of gait events.

To investigate gait cycles, the gait characteristics of both left and right feet were synthesized as shown in Figure 8 based on the aforementioned leading clearance matching synchronization mechanism. By observing Figures 1, 3, and 8, a gait cycle of the right foot starts from a TO event (TO_R_) and then passes through the characteristics FA_R_, TV_R_, and IC_R_, to complete the swing phase of the right foot. Subsequently, the right foot turns into the stance phase, which has the characteristic sequence of an OTO event (i.e., TO_L_), HR_R_, and an OIC event (IC_L_). For a gait cycle of the left foot, the sequence of gait characteristics is the same as that of the right foot.

In Figure 3, the easiest temporal separation positions to be recognized for the two gait phases are the TO event (a signal peak) and the IC event (a zero-crossing point) between the V-IC point (a signal valley) and the IC-P point (a signal peak). Therefore, if the signal characteristics of TO and IC were identified exactly, then gait phases and cycles could be segmented accordingly. The temporal positions of the above two characteristics could then be used to compute the durations of a stance phase, a swing phase, and the associated gait cycle.

Since it is a tedious work to find out all gait events manually from the recorded video, computer algorithms should be considered to extract the gait characteristics from the triaxial accelerometrical signals. Since the TO event and the zero-crossing point for IC are the most important characteristics to segment and extract all gait characteristics, the detailed working steps for the gait segmentation and characteristic extraction are described in Algorithm 1.

In the GCS algorithm, there are five major steps to locate the temporal positions of V-IC, IC-P, and IC, and then, a gait cycle can be determined. To extract other gait characteristics, a gait events' neighboring relationship shown in Figures 1, 3, and 8 can be found and exploited. For example, the temporal interval between the IC and TO points is about 60% of a gait cycle length; thus, the IC temporal position to the TO should normally be 60%  ±  5%. This neighboring relationship can be utilized to locate the TO temporal position from the IC. The rest characteristics can also be located in the same way as the working steps described in Algorithm 2.

In Algorithms 1 and 2, no working procedure for extracting the HR characteristic was described. This is because no significant signal feature around the HR gait event can be found. Therefore, the temporal positions of the HR event were extracted manually by observing the recorded video under the supervision of the medical doctor in this study.

### 3.4. Correlation Analysis

In fast walking, we are interested in exploring the relationship of tripping risks to the gait cycles/phases/events/periods under a chosen cadence. Through a correlation analysis, we could examine the relationship between the cadence and the gait characteristics, which were obtained by the aforementioned algorithms. Subsequently, the correlation coefficients could be calculated to express the trend of positive, negative, or no correlation between two data sets. Let {*x*_1_, *x*_2_, …, *x*_*n*_} and {*y*_1_, *y*_2_, …, *y*_*n*_} be two data sets for the gait characteristics. Then, Pearson's correlation coefficient *r*_*xy*_ is computed by

(1) $$\begin{array}{c} r_{xy}=\frac{n\sum x_{i}y_{i}-\sum x_{i}\sum y_{i}}{\sqrt{n\sum x_{i}^{2}-{\left(\sum x_{i}\right)}^{2}}\cdot \sqrt{n\sum y_{i}^{2}-{\left(\sum y_{i}\right)}^{2}}}. \end{array}$$

For the interpretation of the correlation coefficient, the positive/negative sign means the trend instead of the degree. The correlation degree between 0 ~ ±0.4, 0.4 ~ ±0.7, and 0.7 ~ ±1.0 indicates that the two data sets have low, moderate, and high correlation, respectively. The correlation degree is ±1.0, which means that the two sets are totally correlated.

## 4. Results

In the walking experiment, there were totally 2600 gait cycles acquired and stored into 175 records. With the segmentation and extraction algorithms, the characteristics listed in Table 1 were obtained by locating their temporal positions. Then, all gait periods and cycles belonging to each participant were normalized by the ratio in percentage corresponding to the beginning of each gait cycle.

### 4.1. Cadence versus Gait Events

The correlation analysis for the cadence and relevant characteristics were calculated as shown in Table 1. In this table, we can find that the most important characteristics correlated to the cadence are the TO and V-IC characteristics (Figure 3), which can be used to delineate the phases of a gait cycle. Note that the V-IC point is closely corresponding to the IC event. The correlation coefficients of the TO and V-IC to the cadence are −0.74 and +0.86, respectively. Thus, the higher the cadence is, the larger the motion strength in the terminal swing period (between the TV and IC events) will be applied and, also, the smaller the motion strength in the TO event due to the negative correlation.

In Table 1 and Figure 8, the FA and OHR (opposite HR) characteristics, which are corresponding to the FA and opposite HR events, show the relevant correlation with the cadence. These two characteristics can be corresponded to the gait periods of the midswing and the opposite terminal stance with the correlation coefficients of −0.64 and −0.72, respectively. It indicates that the higher the cadence is, the shorter the two periods will be.

Basically, the normal walking speed in daily life of a person is equal to the product of his/her step length and the cadence. Since the average step length in walking of a person is highly related to his/her height, the walking speed is mostly affected by the cadence. In the order of absolute values of the correlation coefficients, the cadence variation in walking is mainly affected by the characteristics of V-IC (related to the TV and IC events, or the terminal swing period), TO, and OHR (or the opposite terminal stance period). For example, the signal curve around V-IC changes prominently in the terminal swing period because the swinging foot conducts greater acceleration and deceleration in fast walking. When walking slowly, the situation becomes contrapositive.

From the above analysis, it can be easily found that higher cadences trigger larger motion strength of the swinging gait events and shorten the action time in the terminal swing and opposite terminal stance periods. This situation can likely raise the tripping risks provided that an accident hinders the progression of continuous gait events in the swing phase.

### 4.2. Cadence versus Gait Periods

**Figure 9:**
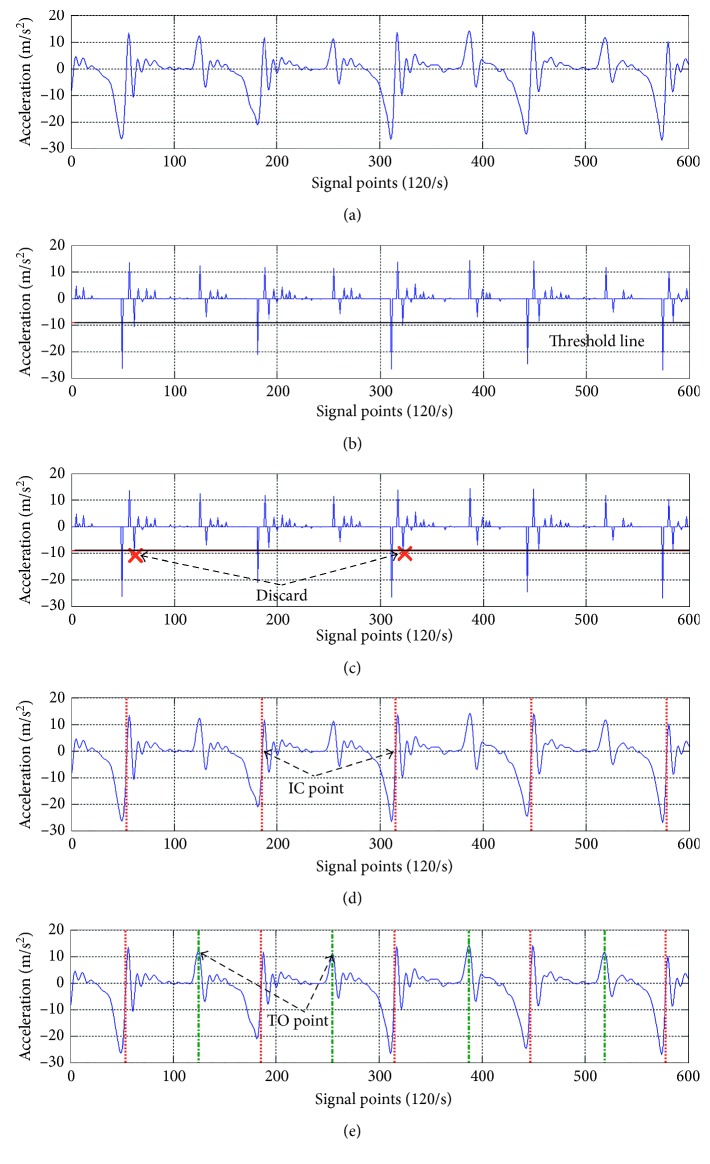
Gait signals processed by the gait cycle segmentation algorithm: (a) filtered signals, (b) setting up a threshold line, (c) unqualified valley points that are discarded, (d) “IC” finding, and (e) “TO” finding.

In Figure 9, the V-IC characteristic acts as an anchor point for segmenting each gait cycle. We analyzed the correlation of the cadence and gait periods for each gait cycle and obtained the average correlation coefficients as listed in Table 2. In this table, the correlation coefficient of “No_cue” was obtained based on playing a music tempo without reminding the participants to notice the tempo, and for “Cue,” the participants were informed to follow the music tempo. Obviously, the music tempo influences slightly the swing phase of every gait cycle in the “No_cue” case, since the beats can still influence the participants by hearing. In “Cue,” every gait period was influenced greatly by different musical rhythms so that the tempo changed the gait cycles and produced different walking speeds.

### 4.3. Gait Phase Variation versus Walk Types

Since the music tempos could greatly affect gait cycles, the gait phase separation on both feet was done with Algorithms 1 and 2 as shown in Table 3, where “Normal” means walking comfortably and naturally; NC_80, NC_100, and NC_120 mean walking in the “No_cue” cases with tempo 80, 100, and 120 BPM; and C_80, C_100, and C_120 mean walking in the “Cue” cases with tempo 80, 100, and 120 BPM.

As walking in “Normal” and “No_cue,” the gait phases of both feet differ only around the range of less than 1.22%. For walking in “Cue,” the difference in C_80 and C_100 is small (≤0.33%), but it becomes large (=1.67%) in C_120. In addition, the gait phase variation increases as the cadence increases in “Cue.”

In Table 3, it can be seen that the left foot spends longer time in the swing phase and shorter time in the stance phase in comparison with the right foot. It reveals that the participants would like to match the beat with the left IC gait event so that the gait cycles of the right foot tend to vary greatly to minimize the speculation of the next beat. In addition, there is no obvious tendency of the gait phases in the cases of “Normal” and “No_cue.” However, in “Cue,” a greater swing time variation happens between C_100 and C_120. It shows an increasing tendency that the left foot spends longer time in swing.

**Figure 10:**
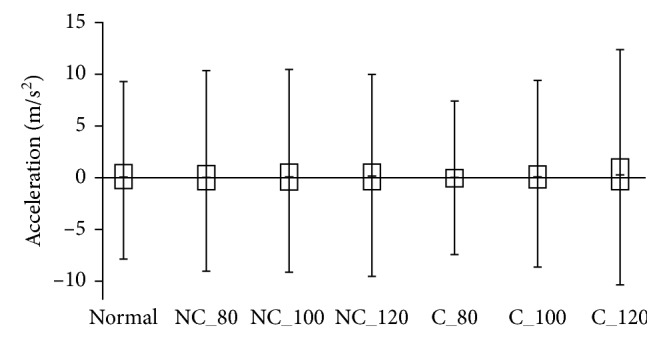
The box plot of the gait cycle variations in *y*-axis.

To disclose more messages from the above observations, we examine the gait signals on the *y*-axis, which can reveal the situations of left and right body sways, as shown in Figure 10. Note that the gait signals in acceleration on *y*-axis in Figure 10 are corresponding to the gait signals on *x*-axis in Table 3. Since the gait cycle (particularly in stance) and body sway have a close relationship to the balance, the extreme values of acceleration on *y*-axis can be used as a balance indicator for the fast walk. Thus, the walking balance in gait is investigated according to different walking speeds based on with and without musical cue. In Figure 10, a box plot shows an interval between a pair of acceleration's maximum and minimum and an interquartile range of acceleration for one walk type.

According to the walk types, we can see that the box plots for the types in “No_cue” are almost similar to each other, and for “Normal,” its box plot is a little smaller to the cases of “No_cue.” This means that walking comfortably and naturally and walking without caring about the cue may have stable and balanced movements in gait with the lower body sway. As walking in “Cue,” the increasing tendency of the body sway matches the increase of cadence. Especially, the case of C_120 is the most unstable and unbalanced in all cases due to the large range of acceleration from the left and right body sways. In addition, the case of C_80 is the most stable and balanced in all cases since the low cadence regulated by the slow tempo benefits the balance control of body's gait movements.

### 4.4. Gait Periods versus Walk Types

In Table 4, the percentage of each left/right gait period is calculated with respect to each gait cycle occurring in the seven different walk types. Noteworthily, the gait periods of all participants are consistent after the normalization of various gait cycles. In this table, we can see that the walk types of “Normal” and “No_cue” have no big difference in the seven gait periods since the walking cadence was determined by the participants. For the walk types of “Cue,”, it can be observed that the gait periods of the left/right preswing and loading response are not sensitive to the higher cadence variation (i.e., 100 and 120 BPM) but is sensitive to the slow tempo such as 80 BPM.

For the gait periods of the initial swing, midswing, and midstance of both feet, their period lengths in percentage increase as the cadence increases. For the terminal swing and terminal stance, their period lengths in percentage decrease as the cadence increases. Thus, we can find that the musical rhythm can intervene with the walking speed by regulating the cadence, which will affect the increase or decrease of gait period lengths.

In addition, we find that the increments of the gait period lengths for the midstance, midswing, and initial swing are more than those in all other cases, and the same situation happens for the decrements of the period lengths for the terminal swing and terminal stance. This indicates that there exists a tripping risk: the longer time for doing the midswing means the stride becomes bigger, and the shorter terminal swing means the movements in the tibia vertical to knee extension (before the initial contact) become faster.

If the walking paths were not flat and there were bumps that might block the movements in the tibia vertical to knee extension, a tripping risk would raise greatly due to the longer time in midstance blocking the next stance support while the center of body mass is moving ahead of the next stance support point. In summary, larger steps or strides in walking maintain larger gait speed and consume more calories. In this way, cadence can be kept slower to prevent tripping risks, but we still have higher gait speed to consume calories and promote health.

## 5. Conclusion

Due to technological and spatial limitations, fast walking for long distance in daily life is difficult to be observed by scientific equipment. In the literature, most of the gait experiments were done by walking on treadmills [5, 7, 8, 10]. Actually, gait signals acquired on treadmills are quite different from the signals on the ground. This hinders the observation of a fast gait on ordinary ground. In this study, we conducted all the walking tests in a flat and straight pathway, intended to reveal important clues for tripping risks in fast walking.

In the walking tests, music cueing in three tempos were employed to regulate the walking speed of participants, and seven walk types, which include one “Normal,” three “No_cue,” and three “Cue” tests, were devised for the acquisition of gait signals. From the extracted gait characteristics, we found that “V-IC” is an important sign that has the largest signal variation in one gait cycle and is highly correlated with cadences. Thus, it can be used to compute the cadence of one specific walk.

In the “Normal” and “No_cue” tests, the difference in cadences, gait phases, and gait periods in both types is minor since the background music tempos in “No_cue” are mostly ignored by the participants. Meanwhile, the left and right body sways in the frontal plane are insignificant. It means that the gaits are stable and balanced in body movements.

In the “Cue” tests, every gait period is influenced greatly by different music tempos, which may cause various walking speeds. Firstly, a larger time variation in foot swing exists between the cadences in 100 and 120 beats. By observing the gait period's variation in the midswing and terminal swing, the longer midswing time means the bigger stride, and the shorter terminal swing time indicates the faster movements in the tibia vertical to knee extension. Secondly, by the observation to a higher cadence (e.g., 120 beats), another factor of tripping risks may exist due to the high negative correlation of midstance and midswing to the higher cadence. This condition causes smaller foot clearance over the ground and increases greatly the probability of tripping. As walking through ordinary or uneven ground, the tripping risk of passing over bumps becomes substantial in faster movements.

A walking speed is determined from a cadence multiplied by a stride length, and it indicates that a bigger stride length needs only a lower cadence to keep the same walking speed. Therefore, in the clinical practice, the principle of fast walking safely in the outdoors should be based on a lower cadence with bigger strides for health promotion. Remarkably, the aforementioned results show that the cadence below 120 beats is preferred. From another point of view, a completely flat ground becomes a necessary and sufficient condition for fast walking. The principle can also apply to indoor activities or locomotion since our experimental ground is close to a home environment.

## Figures and Tables

**Algorithm 1:**
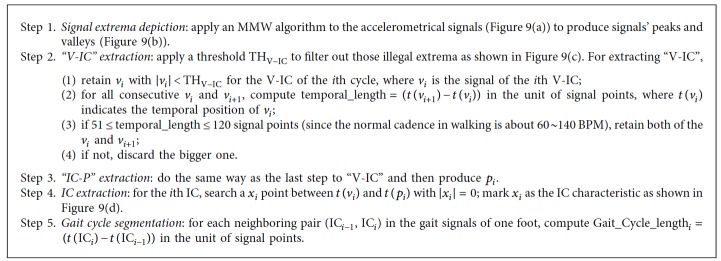
Gait cycle segmentation (GCS).

**Algorithm 2:**
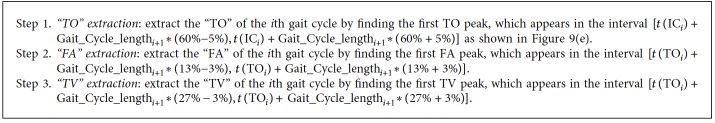
Gait characteristic extraction (GCE).

**Table 1:** Correlation analysis of the cadence and relevant gait characteristics.

	Cadence	TO	FA	OHR	TV	V-IC	IC-P
Cadence	1	−0.74	−0.64	−0.72	−0.32	0.86	0.03
TO	−0.74	1	0.75	0.59	0.28	−0.72	0.27
FA	−0.64	0.75	1	0.76	0.18	−0.52	0.04
OHR	−0.72	0.59	0.76	1	0.54	−0.73	0.25
TV	−0.32	0.28	0.18	0.54	1	−0.52	0.19
V-IC	0.86	−0.72	−0.52	−0.73	−0.52	1	−0.29
IC-P	0.03	0.27	0.04	0.25	0.19	−0.29	1

**Table 2:** Correlation analysis of the cadence and gait periods.

Period	Type	
	No_cue	Cue
Preswing	0.10	0.82
Initial swing	−0.10	−0.83
Midswing	−0.22	−0.79
Terminal swing	0.20	0.82
Loading response	−0.02	0.82
Midstance	0.05	−0.86
Terminal stance	−0.03	0.84

**Table 3:** Percentage of the left/right gait phase with respect to the normalized gait cycle in seven different walk types.

Phase (%)	Type						
	Normal	NC_80	NC_100	NC_120	C_80	C_100	C_120
Left swing	42.22	42.00	41.67	42.00	38.00	41.22	43.00
Right swing	41.00	41.78	40.67	41.33	37.89	40.89	41.33
Left – right	1.22	0.22	1.00	0.67	0.11	0.33	1.67
Left stance	57.78	58.00	58.33	58.00	62.00	58.78	57.00
Right stance	59.00	58.22	59.33	58.67	62.11	59.11	58.67
Left – right	−1.22	−0.22	−1.00	−0.67	−0.11	−0.33	−1.67

**Table 4:** Percentage of the left/right gait periods with respect to each gait cycle occurring in the seven walk types.

Period (%)	Type						
	Normal	NC_80	NC_100	NC_120	C_80	C_100	C_120
Left preswing	9.51	9.09	9.97	8.86	11.21	9.57	9.35
Left initial swing	13.00	13.22	12.78	13.22	11.33	13.56	14.11
Left midswing	12.89	12.22	13.78	13.78	11.00	13.78	15.00
Left terminal swing	16.33	16.56	15.11	15.00	15.67	14.89	13.89
Left loading response	9.27	9.13	9.69	8.80	10.90	9.32	9.32
Left midstance	19.89	19.56	20.11	19.78	18.11	19.22	21.33
Left terminal stance	19.11	20.22	18.56	20.56	21.78	19.67	17.00
Right preswing	9.19	9.21	9.86	9.40	11.16	9.37	9.30
Right initial swing	13.78	13.33	13.00	13.00	10.78	12.44	15.00
Right midswing	14.44	13.89	13.89	14.44	11.22	13.33	14.56
Right terminal swing	12.78	14.56	13.78	13.89	15.89	15.11	11.78
Right loading response	9.59	9.01	9.80	9.26	10.95	9.52	9.36
Right midstance	19.89	19.33	19.11	20.00	18.78	19.44	21.78
Right terminal stance	20.33	20.67	20.56	20.00	21.22	20.78	18.22

## References

[1] Abbud G., Li K., DeMont R. (2009). Attentional requirements of walking according to the gait phase and onset of auditory stimuli. *Gait and Posture*.

[2] Tsuruoka M., Shibasaki R., Tsuruoka Y. Analysis of 1/f fluctuations of walking listening to Mozart’s music.

[3] Delval A., Moreau C., Bleuse S. (2014). Auditory cueing of gait initiation in Parkinson’s disease patients with freezing of gait. *Clinical Neurophysiology*.

[4] Bourgeois A. B., Mariani B., Aminian K., Zambelli P., Newman C. (2014). Spatio-temporal gait analysis in children with cerebral palsy using, foot-worn inertial sensors. *Gait and Posture*.

[5] Nascimento L. R., de Oliveira C. Q., Ada L., Michaelsen S. M., Teixeira-Salmela L. F. (2015). Walking training with cueing of cadence improves walking speed and stride length after stroke more than walking training alone: a systematic review. *Journal of Physiotherapy*.

[6] Roerdink M., Bank P. J., Peper C. L. E., Beek P. J. (2011). Walking to the beat of different drums: practical implications for the use of acoustic rhythms in gait rehabilitation. *Gait and Posture*.

[7] Mendonca C., Oliveira M., Fontes L., Santos J. (2014). The effect of instruction to synchronize over step frequency while walking with auditory cues on a treadmill. *Human Movement Science*.

[8] Eikema D., Forrester L., Whitall J. (2014). Manipulating the stride length/stride velocity relationship of walking using a treadmill and rhythmic auditory cueing in non-disabled older individuals. A short-term feasibility study. *Gait and Posture*.

[9] Wittwer J. E., Webster K. E., Hill K. (2013). Music and metronome cues produce different effects on gait spatiotemporal measures but not gait variability in healthy older adults. *Gait and Posture*.

[10] Byung-Woo K., Lee H.-Y., Song W.-K. (2016). Rhythmic auditory stimulation using a portable smart device: short-term effects on gait in chronic hemiplegic stroke patients. *Journal of Physical Therapy Science*.

[11] Mariani B., Rouhani H., Crevoisier X., Aminian K. (2013). Quantitative estimation of foot-flat and stance phase of gait using foot-worn inertial sensors. *Gait and Posture*.

[12] Khurelbaatar T., Kim K., Lee S., Kim Y. H. (2015). Consistent accuracy in whole-body joint kinetics during gait using wearable inertial motion sensors and in-shoe pressure sensors. *Gait and Posture*.

[13] Rubenstein L. Z. (2006). Falls in older people: epidemiology, risk factors and strategies for prevention. *Age and Ageing*.

[14] Lord S., Sherrington C., Menz H., Close J. (2007). *Falls in Older People: Risk Factors and Strategies for Prevention*.

[15] Whittle M. W. (2007). *Gait Analysis: An Introduction*.

[16] Nordin M., Frankel V. H. (2001). *Basic Biomechanics of the Musculoskeletal System*.

[17] Magee D. J. (2014). *Orthopedic Physical Assessment*.

[18] Texas Instruments (2015). *MSP430F552x, MSP430F551x Mixed-Signal Microcontrollers*.

[19] STMicroelectronics (2011). *LIS3DH: MEMS Digital Output Motion Sensor*.

[20] Sony (2014). *Digital HD Video Camera Recorder Handbook*.

[21] Monson I. (2009). *Saying Something: Jazz Improvisation and Interaction*.

[22] Rao K. R., Kim D. N., Hwang J. J. (2010). *Fast Fourier Transform–Algorithms and Applications*.

[23] Electronics Hub (2015). *Butterworth Filter*.

[24] Bruce L. M., Adhami R. R. (1999). Classifying mammographic mass shapes using the wavelet transform modulus-maxima method. *IEEE Transactions on Medical Imaging*.

[25] Erer K. S. (2007). Adaptive usage of the Butterworth digital filter. *Journal of Biomechanics*.

